# Supplementary material for: Differential Drug Susceptibility across Trichomonasvirus Species Allows for Generation of Varied Isogenic Clones of Trichomonas vaginalis
Source: Pathogens. 2024 Aug 29;13(9):733. doi: 10.3390/pathogens13090733 (PMC11434947; doi:10.3390/pathogens13090733)
Supplement: Supplementary file 1 [file pathogens-13-00733-s001.zip › pathogens-3177234-supplementary.pdf]

Table S1. Primers used for RT-qPCR

Target	Forward Primer Sequence	Reverse Primer Sequence
Actin	TCACAGCTCTTGCTCCACCA	AAGCACTTGCGGTGAACGAT
TVV1-JH37A#2	ATTAGCGGCGTTTGTGATGCA	CCTGGGGTTTGCGTTCCTTG
TVV1-JH191A#4	ATTAGCGGTGTTTGTGATGCA	TTGCCATGCTCTAGCTTGCG
TVV3-JH32A#4	GAAGCTGAGCTTCTCGTCACAG	ATGAGGTTGGACAGACTTCCTGTC
TVV1-JH162A#4	ATTAGCGGTGTTTGTGATGCA	TTGCCATGCTCTAGCTTGCG
TVV2-JH162A#4	CTGACTTACACCGACAGTTGGAC	GTCTTTTAAGAAAGCATCGTTGCGAC
TVV3-JH162A#4	GATTGGTGCATCGCTAGCATTG	TTGGTTGCCACTCCCATGATG
TVV5-JH162A#4	TCGTCTCTGTCTAGCTGCCTCT	CGTTCTTGCACCAGAATGGTGATG
TVV1-RU357	ATTAGCGGCGTTTGTGATGCA	ACTTGAGGCTTGCATTCCTTGAG
TVV2-RU357	CTGACTTACACCGACAGTTGGAC	GTCTTTTAAGAAAGCATCGTTGCGAC
TVV4-RU357	GCCGACTTGAAGGTCAACTGC	GTGTAGATAGTTCTTATGGCGAGACGC
TVV5-RU357	CCTATATGCTCGTCTCTGTCTGGC	GAATGGACGTGGTCAGTGAAACTG

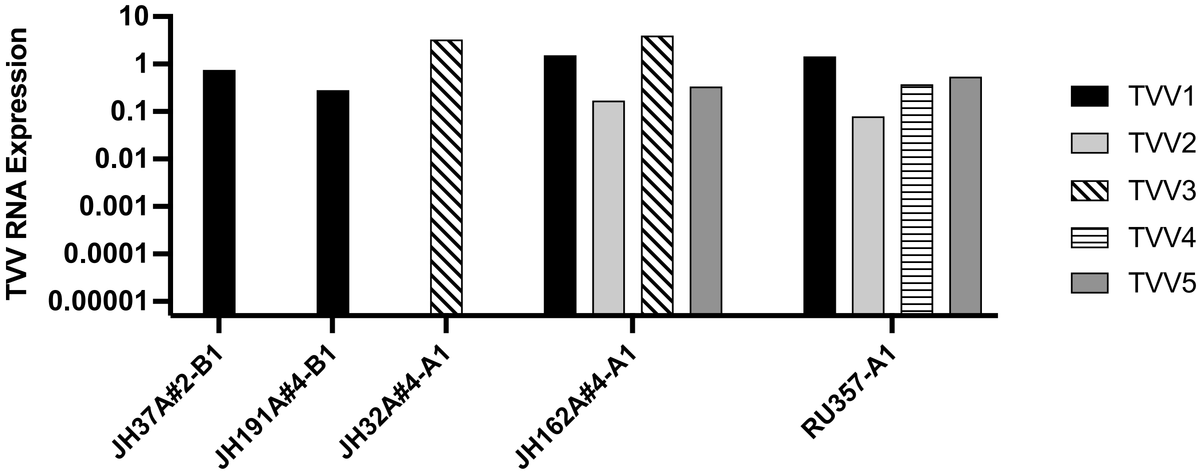

Figure S1. Validation of the presence of trichomonasviruses in isolates tested. RNA was extracted from each culture and screened for the presence of virus by RT-qPCR as described in section 2.1.

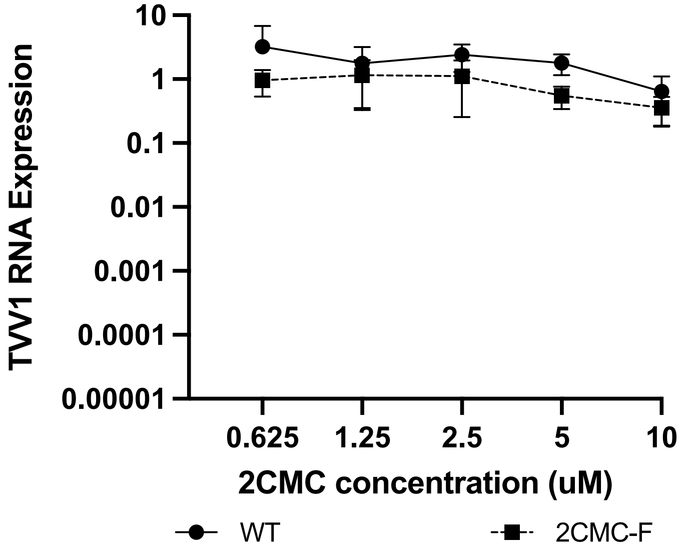

Figure S2. Susceptibility to 2CMC is not significantly different between TVV1 in parent isolate JH37A#2-B1 and TVV1 in 2CMC-treated but uncured clone JH37A#2-B1-2. Cells from parent isolate JH37A#2-B1 and clone JH37A#2-B1-2 were incubated in media containing 0–10 µM 2CMC for 24 h. Relative viral RNA abundance in each sample was quantified and displayed as described for Figure 2.
